# 

**Published:** 1829-06

**Authors:** 


### MONTHLY LIST OF MEDICAL BOOKS.

[*Medical Works cannot be entered on this List except a copy be sent for the purpose; the titles of Books having frequently been transmitted to us, as published, which have not appeared for weeks, or even months, after.*]

Medical Botany; or Illustrations and Descriptions of the Medicinal Plants. By JOHN STEPHENSON, M.D. F.L.S. and J. M. CHURCHILL, F.L.S. &c.—Published by Tilt, Fleet street.

These Numbers of this excellent and very useful work deserve the same degree of approbation which we have bestowed upon the preceding parts. The two plates of the Aloes are particularly valuable. The Aloe Vulgaris, of which the plate is given in No. 27, has appeared, we believe, in no other work excepting SIBTHORP's Fl. Græca, the price of which is fifty guineas, and of which seven copies only have been published. The Aloe Socotrina has been taken from a living specimen which blossomed this spring. The plate of Ergot is also valuable, and well executed. The account of the poisonous and medical effects of this now important remedy is highly interesting and instructive. Several other plates are contained in these Numbers, together with correct descriptions of them in the text.

Medical Report of the House of Recovery and Fever Hospital, Cork street, Dublin, for the year 1827. By JOHN O'REARDON, M.D. &c.—Dublin.

An interesting and practical document, from which we shall select extracts.

A Practical Dissertation on the Waters of Leamington Spa; including the History of the Springs; a new Analysis of their gaseous and solid Contents; the Rules for drinking the Waters, Bathing, Diet of the Patients, and other Regimen. By CHARLES LOUDON, M.D.—8vo. 1828.

The author of this Dissertation has very satisfactorily effected the object he has had in view, of giving the profession a monograph to refer to, that they may be able to judge of the nature of the mineral springs at Leamington, and of the various ways in which they are used.

Twenty-fourth Annual Report of the London Ophthalmic Infirmary for curing Diseases of the Eye, founded by J. C. SAUNDERS.—London, 1829.

The committee of this well-known Infirmary made two very gratifying statements on the twenty-fifth anniversary of the institution: the funds are much improved, and the number of patients relieved greatly increased.

Elements of Medical Statistics; containing the Substance of the Gulstonian Lectures delivered at the Royal College of Physicians; with numerous Additions, illustrative of the Comparative Salubrity, Longevity, Mortality, and Prevalence of Diseases in the principal Countries and Cities of the Civilized World. By F. Bisset Hawkins, m.d. of Exeter College, Oxford, &c.—8vo. pp. 234. Longman and Co. London.

Observations on the Phrenological Development of Burke, Hare, and other atrocious Murderers; Measurements of the Heads of the most notorious Thieves in various Prisons. Presenting an extensive series of Facts, subversive of Phrenology. Read before the Royal Medical Society of Edinburgh. By Thomas Stone, Esq. President of the Royal Medical Society.—8vo. pp. 75. 1829.

### NOTICES.

*Anonymous reviews of books, whether favorable or unfavorable, can never be admitted into this Journal. The Editors must also take the liberty of observing, that the attempt to procure the publication of such articles is not creditable to the writers of them.*

Mr. Broughton's *Communication has been received.*

ERRATUM *in the last Number.* The last line in p. 442 to be at the top of p. 439.

### INDEX

TO THE

SIXTH VOLUME OF THE NEW SERIES

OF THE

### London Medical and Physical Journal.

PAGE

ABDOMEN, wounds of, for the purpose of suicide . 423
Abortion, Dr. Granville's work on, 280
Abscesses, chronic, in the female breast . . . 327
Acupunctuation in India . 171
Affidavits, false, made at Apothecaries' Hall . . . 279
Ague treated by external use of Quinine . . . 557
Alum, use of, in Colica Pictonum 95
Ammonia, Acetate of, in uterine diseases . . . 270
Amputation, superiority of making one incision through the integuments and muscles, in 80
Anatomy, analysis of Dr. Craigie's Elements of General and Pathological . . . 243
——, Mr. Tuson's Compendium of . . . 472
——, Mr. Guthrie's Letter to the Secretary of State, containing remarks on the Report of the Select Committee of the House of Commons on . 260
Aneurism of the abdominal aorta, 553
——, axillary, case of, caused by reduction of a luxated humerus . . . 314
——, Dupuytren's case of femoral . . . 173
—— of the carotid, cured by Valsalva's plan . . 273
——, spontaneous cure of femoral, aided by pressure . 413
Angina pectoris without organic affection of heart or large vessels . . . 353
Animal Magnetism, Mr. Chenevix on . . . 219, 491

PAGE

Aorta, case of contraction of, in which the vessel was nearly impervious . . . 240
——, aneurism of abdominal . 553
—— descendens, rupture of . 290
Apoplexy, disposition to, in old age . . . . 257
——, case of pulmonary . 359
Apothecaries, regulations of the 469
——' Hall, false affidavits made at, by students . 279
Arabian mode of treating fractures . . . . 175
Arsenic in sulphur, method of detecting . . . 564
Ash, Dr. death of . . 473
Asthma, on the use of the Lobelia Inflata in . . . 558
Axillary aneurism, case of, caused by reduction of luxated humerus, 314

Bath, Dr. Ridgway on the origin of the Vapour . . 215
Belladonna externally applied, and reducing strangulated hernia . . . 558
Blistering never serviceable in mania . . . 74
Blisters injurious in delirium tremens . . . 15
Blood, fluidity of, after death 466
——, appearances of, how far they show necessity for bleeding . . . . 550
——, experiments on the velocity of the circulatory motion of the . . . 357
Bloodletting in insanity, Dr. Burrows on . . . 69
Borax useful in diseases of the skin . . . . 557

PAGE
Bonaparte, opinion of a French physician as to the death of . 354
Botany, Pupil's Introduction to, by Dr. Steggall . . 281
———, plan of Mr. Burnett's Lectures on . . . 566
Brain, abscess in the, cured by the trephine . . . 452
Breast, hydatid disease of female, diagnosis of . . . 328
———, effect of common inflammation in, by Sir A. Cooper, Bart. . . . 325
———, Sir A. Cooper on the irritable tumor of the female . 337
———, Illustrations of Diseases of, by Sir Astley Cooper, Bart. 323
———, ecchymosis of the female, 337
———, on the scrofulous swelling of the female . . 334
———, female, on the large and pendulous tumor of . . ib.
Broom seeds as a substitute for coffee . . . 180
Bronchotomy twice performed with success, in cases of laryngitis . . . . 234
Buffy coat of blood not a proof of the propriety of venesection, 550
Bulimia, cases of . . 267, 553
Burns, treatment of, by flour . 297

Calculi removed from the bladder by urethral forceps . 519
Calculus impacted in the neck of the bladder and prostatic portion of the urethra . 237
———, case of lachrymal . 78
Cambridge medical degrees, new regulations respecting . 377
Camphor in puerperal mania . 557
Cancerous tubercles in different parts of the body . . 134
Cancer, term applied to all swellings of the breast by uninformed surgeons . . . 324
Carotid artery, aneurism of, cured by Valsalva's plan . 273
Catarrhus vesicæ of old persons, remarks on . . . 401
Cervical vertebræ, fractures of 49
Chemistry unable to detect the processes carried on in the animal and vegetable economy . 179
Cheonopodium olidum, recommended as an emmenagogue by Mr. Houlton . . 278
Chest, affections of, after injuries and operations . . 318
———, on diseases of, by Laennec 338
Chest, wounds of, for the purpose of suicide . . . 423
Chlorine, preparation of grains and seeds by . . 180
Cholera, analysis of Dr. Christie's work on . . . 159
Coffee, new kind of . . 180
Colica pictonum, treatment of by alum . . . 95
Conception, case of double, with double uterus . . 178
Concussion of the brain . 38
Contusions, appearances of, inflicted after death . . 464
Convulsive epidemic, symptoms of, 79
Convulsions, remarks on infantile, from spasm of the intestines . . . . 393
Cooper, Sir Astley, Illustrations of the Diseases of the Breast 323
———, Bransby, Esq. vase presented to him by his pupils . 567
——— v. Wakley, trial of . 83
Cornea, Dupuytren's treatment of spots on . . . 562
Corpuscles in the eye, case of . 121
Cough, on the effects of a shock of water in spasmodic . 458
———, case of obstinate, cured by removal of elongated uvula 559
Cranium, fracture of, followed by depositions of lymph in the lung and on the pleura . 40
Crystalline lens, on the reproduction of . . . 449
Cuticle, exfoliation of . . 556

Death from the prick of a pin 462
Degrees, new regulations with regard to the Cambridge medical . . . . 377
Delirium senile, Burrows on . 65
——— tremens, case of . 551
———————, Mr. Smith on 9
———————, Dr. Burrows on 61
Demerara, on diseases in, by Mr. Barcome . . 283
Diabetes successfully treated . 301
Diamond, discovery of the mode of making the . . 181
Digestion, Dr. P. Wilson on the function of . . . 547
Digitalis combined with quinine in consumption . . 557
Dissection, petition of the Westminster Medical Society on the supply of subjects for . 183
———, Mr. Guthrie's letter on the supply of subjects for 260
Duodenum, perforation of the 43

PAGE
Duodenum, ulceration and perforation of . . . 76
Dysentery, Dr. Speers on, in the Essex prison-hulk . . 199

Ear, effects of the section of the semicircular canal of, in birds 76
Ecchymosis of the female breast 337
Emmenagogue, Cheonopodium olidum used as an . . 278
Endermic method of using remedies . . . 308
Epidemic of Gibraltar . . 82
Ergot of rye in cases of leucorrhœa, by Dr. Marshall Hall . 379
Eruptions, artificial, in mania . 74
Erysipelas phlegmonodes treated by compression . . 81
——— cured by compression . . 367
Essex prison hulk, Dr. Speers on the diseases on board the, in 1825-6-7 . . . 197
Exfoliation of the bones of the pelvis, causing obstinate sinuses 410, 559
Exhalations, innocuous nature of putrid . . . 277
Extra-uterine fœtation, case of, by Mr. Jewel . . 36
——— abdominal pregnancy, case of . . 275
Eye, case of fungus of . . 141
—— case of corpuscles in . 121
Eyes, inflammation of the . 1

Face, tumor successfully extirpated from . . . 462
Fallopian tubes, obstruction of 452
Flour as a remedy for burns . 297
Femoral aneurism, spontaneous cure of, aided by pressure . 413
Femur, union of the fracture of the neck of the . . 368
Fœtation, remarkable case of preternatural, in which there was neither anus, funis, nor umbilicus . . . 166
———, case of extra-uterine, by Mr. Jewel . . 36
Fœtus, functions of the intestinal canal and liver in, by Dr. Lee 356
———, nourishment of, in the womb . . . 167
Forbes, Dr. translation of Laennec on Diseases of the Heart . 339
Foundling Hospital, Mr. Perry's appointment of surgeon to . 471
Fractures, Arabian mode of treating . . . 175

PAGE
Fruits, employment of slates for hastening the maturation of . 180
Fumigations, remarks on mercurial . . . 384
Fungus hæmatodes, case of . 368
——— cured by alum and the red oxyd of mercury 273
——— of the eye, case of . 141

Gastrotomy, pretended operation for, in the case of a man who fancied he had a serpent in his belly . . . 450
Gibraltar, epidemic of . . 82
———, review of Mr. Fraser's Letter on the Febrile Distempers of . . . 153
Gleet cured by injection of seawater . . . 275
Grain prepared by chlorine . 180
Guthrie, G. J. Esq., introductory lecture at the College of Surgeons . . . 373
——— on Inflammation of the Eyes . . . 1
Gyration and swinging in insanity 71

Head, injuries of . 38, 41
Heart, atrophy of the . . 349
——— hypertrophy of auricles of, 340
——— fatty degeneration of . 350
——— polypi of, and large vessels . . . . 351
——— rupture of . . 548
——— wound of, (patient lived one hour) . . . 414
——— diseases of, caused by onanism . . . 451
——— malformation of . 548
——— extraordinary growth of 547
——— on feigned diseases of, from use of hellebore . 564
——— causes of ossifications of 343
——— softening of muscular substance of . . . 347
——— induration of the muscular substance of . . ib.
——— on the changes produced by diseases of, in the texture of other organs . . 342
——— inflammation of inner membrane of, very rare . 351
——— Laennec on Diseases of 338
——— malformations of . 349
Hellebore causing apparent disease of the heart . . 564
Hemorrhage, remarkable predisposition to . . . 554
——— from the mammæ, case of habitual . . 555

PAGE
Hemorrhage, efficacy of tannin in suppressing uterine . 456
Hennen, Dr. death of . . 185
Herbivorous habit in an idiot . 276
Hernia, remarks on obstructed 425
———, necessity of separating all the adhesions of intestines in operating for . . 429
———, operation for, may be necessary, without symptoms of strangulation . . 435
———, on inflamed . . 430
———, treatment of . . ib.
———, propriety of purging after operation for . . 426
———, Mr. Stephen's proposal for the radical cure of . 436
———, analysis of Mr. Stephens on . . . . . 425
———, case of strangulated crural, which, after gangrene had supervened, was cured by an artificial opening of the tumor . . . 123
———, strangulated, reduced by external use of belladonna . 558
——————— umbilical, (operation performed fifteen days after first symptoms of) 321
———, case of strangulated, (six inches of intestine removed) 560
Herpes, Dupuytren's treatment of corroding . . . 562
Hindostan, on the Diseases in, by Mr. Walsh . 25, 102, 207
Hip, presentation of the, in six successive labours in the same woman . . . 463
Hopkins, Dr. medals given by 471
Hybrid, case of curious . 467
Hydatid disease of female breast, diagnosis of . . . 328
Hydrocyanic acid, utility of cold affusions in cases of poisoning by . . . . . 387
Hydrophobia, no effects from the inoculation with the saliva in cases of . . . 552
Hydrophobia, case of . . 169
Hypertrophy of auricles of heart 340
————— of ventricles of the heart, signs of . . 345
Hysteria, acupunctuation in . 171

Iliac artery, ligature of the external . . . 273
—— vein, obliteration of . 453
Insanity, analysis of Dr. Burrows on . . . . . 61
Insects, remarks on the Leaf . 467

PAGE
Intestines, sudden protrusion of all the, into the scrotum . 415
Intestine, six inches of, removed in case of strangulated hernia 560
Intoxication, efficacy of liquor ammonia in . . . 79
Iron, on the presence of, in tin 464
Irritable tumor of the female breast, Sir A. Cooper on . 335
Ischuria treated by cold affusions 553
————, singular case of . 515
Italy, state of Medicine in . 475
Jurisprudence, analysis of Mr. Forsyth's work on Medical . 541

Knee-joint, case of diseased . 291

Lachrymal calculus, case of . 78
Lambert, Mr. expelled from the Westminster Medical Society 184
Laryngitis, bronchotomy successfully performed in two cases of 234
Larynx, disease of . . 312
———, abscess of; unexpected death . . . 514
Larvæ of insects and other animals found in the stomach and intestines . . . . 151
Leeches, preservation of . 276
———, disease communicated by ib.
Leg, fracture of, communicating with the ankle-joint . . 47
Lens, on the reproduction of the crystalline . . . 449
Leucorrhœa, ergot of rye in cases of . . . 379
Lime, chloride of, in sloughing sores . . . 561
——, universal diffusion of, in natural bodies . . 179
Liston, Mr. of Edinburgh, denies that he is the author of Lectures on Aneurism attributed to him by the Lancet . 279
Lithotomy twice performed in one patient on the same day 237
———— performed twice in three days on the same patient, by M. Dupuytren . 21
—————, difficult case of, operation . . . 130
—————, Bransby Cooper's relation of the case of, the subject of the libel in the Lancet 127
Lithotritie successfully performed 412
Lobelia inflata, on the use of, in asthma . . . 558
London University, prizes distributed at . . . 565
Luke, Dr. death of . . 473

PAGE

Lungs, œdema of, caused by diseases of the heart . 343

Luxations, remarks on the reduction of old . . 318

Magnetism, Mr. Chenevix on Animal . . 219, 491
Mammary tumor, on the chronic, by Sir Astley Cooper . 333
Mammæ, habitual hemorrhage from . . . . 555
Mantis tribe, remarks on the . 467
Measles, eruption of, on one side of the body . . . 553
———, sulphur employed to prevent . . . 269
Medical practice of Italy . 475
Medulla spinalis, alteration of, in a case of tetanus . . 132
Menses, absence of, in a woman in whom the other signs of puberty existed . . 452
Menstruation, case of vicarious 422
——— occurring during pregnancy . . . 55
Mercurial fumigations, remarks on . . . . 384
——— action accelerated by turpentine . . . 501
Mesmerism, Mr. Chenevix on, 219, 491
Midwifery, six hip presentations in the same woman . . 463
———, Mr. Waller's report of cases of . . . 115
———, Mr. Jewel's report of 100 cases of . . 381
———, analysis of Dr. Ryan's Manual on . . . 51
Milk abscess, treatment recommended by Sir Astley Cooper differs from that of Dr. John Clarke . . . . 325
Mushrooms, indications of wholesomeness in . . . 179
Myrtifolia, coriaria, poisoning by the berries of . . 292

Nails, Mr. G. Burnett on diseases of . . . . 91
Napoleon à Saint Hélène, by J. Hereau . . . . 354
Nerves, reunion of the ends of 76
Nervous system, central points of 75
Neuralgia, efficacy of turpentine in . . . . 203
Nipples, Sir Astley Cooper's remedy for sore . . 327
———, remedy for sore . 275
Nitrate of silver, on the use of, in inflammations, wounds, and ulcers, 521

PAGE
Nitrate of silver as a test for vegetable and animal matter . 464
Nympha, Dr. Granville's case of tumor removed by a jugum . 460

Onanism, diseases of the heart caused by . . . 451
Operations succeeded by disease of the chest . . . 318
Opium, Dr. Webster on East-Indian . . . 511
Opiates in insanity . . 73
Ovarian tumor, hair and bones found in . . . 306

Palsy, endermic method of treating . . . . . 309
Paralysis, acupunctuation in . 269
Parturition, analysis of Mr. Ashwell's Practical Treatise on 51
Pathological Anatomy, analysis of Dr. Craigie's Elements of 243
Pelvis, exfoliation of the bones of, causing obstinate sinuses 410, 559
Percussion, use of, to detect organic disease of the heart . 359
Perineum, urinary abscess in the 310
Pericardium, remarkable case of dropsy of . . . 407
Phlebitis after bleeding, Mr. Chinnock's case of . . 176
Phthisis pulmonalis sometimes contagious . . . 441
———, on the direct application of remedies to the lungs in cases of . . 445
———, combination of quinine and digitalis in . . 557
Physicians, first meeting at the College of . . . 278
Physiology, Mayo's Outlines of 280
Pin, suppuration and death from the prick of a . . 462
Placenta, cases of absorption of the . . . . 176
——— expelled four months after delivery . . 563
Pneumo-thorax, Dr. J. Johnson's case of . . . 360
Poisoning by the berries of Coriaria Myrtifolia . . 292
Polypi of the heart and large vessels . . . 351
Pregnancy with cancer of cervix uteri . . . . 563
———, extra-uterine . 50
———, case of extra-uterine abdominal . . . 275
Prize Questions . . 472

PAGE
Prussic acid, utility of cold affusions in cases of poisoning by, 387
Ptyalism, chronic, case of, cured . . . 269
Puerperal insanity, Dr. Burrows on . . . . . 62
——— mania, camphor in . 556
Pulmonary apoplexy, case of . 359
——— Pathology, analysis of Dr. Myddelton's work on 436
Putrid exhalations, innocuous nature of . . . 277

Quinine and digitalis, combination of, in consumption . 557
———, on the external use of ib.
———, adulteration of . 558

Ricini, oleum, use of, causing eruption . . . 458
Rose, Thomas, Esq. death of . 185

Sciatica, endermic method of treating . . . 309
Scrotum, morbid enlargement of 289
———, sudden protrusion of all the intestines into . 415
Sea-sickness, patent granted to Mr. Derbishire for his remedy 184
Secretions, experiments showing the quickness with which they are performed . . 357
Seeds prepared by chlorine . 180
———, preservation of, for vegetation . . . ib.
Seeing in water, on the power of, possessed by certain animals 264
Senile insanity, Dr. Burrows on 65
Silver detected in the internal viscera . . . 467
Skin, borax in diseases of . 557
Sleep in insanity . . 73
Sloughing sores, chlorides of soda and lime in . . . 561
Spasm of the intestines producing infantile convulsions . 393
Spermatic vein, Dr. R. Lee's case of inflammation of . 366
Spinal cord, destruction of portion of . . . 267
Spleen, suppuration of . 77
———, diseases of . . 167
Stomach, case of perforation of 217
———, conformation of the human . . . 166
Stone in the bladder contained in a cyst . . . 131
Strangulation of the intestines, not the only cause demanding the operation for hernia . 436

PAGE
Strictures of the urethra, treatment of, by M. Dupuytren . 35
———, on the division of, by the lancetted stilettes . 535
———, analysis of Mr. Stafford's work on . . ib.
Sudorific medicines, Dr. Holland on . . . . 372
Suicide, Dr. Burrows on . 65
Sulphur, method of detecting arsenic in . . . 564
——— as a preventive of measles, 269
Swinging in insanity . . 71

Tannin, efficacy of, in uterine hemorrhage . . . 456
Tetanus, fatal case of, showing a remarkable alteration of the medulla spinalis . . 132
Textures, on the division of the animal . . . 245
Thigh-bone, union of the fracture of the neck of . . 368
Throat, wound of . . 312
Tin, on the presence of iron in 464
Tubercles, cancerous, case of, in different parts of the body . 134
Turpentine, on the acceleration of the mercurial action by the use of . . . 501
———, efficacy of, in neuralgia . . . 203

Umbilical vein, Dr. R. Lee's case of inflammation of . . 455
Urethra, treatment of strictures of, by M. Dupuytren . 35
Urinary abscess in the perineum, case of . . . 310
Uterine hemorrhage, efficacy of tannin in the suppression of . 456
——— diseases, acetate of ammonia in . . . 270
Uteri, pregnancy with cancer of cervix . . . 563
Uterus, enlargement of, containing a bag full of pus . . 452
———, case of double . . 178
———, excision of . . 174
———, absorption by the . 176
———, on inflammation of the veins of the . . . 267
———, Dr. R. Lee's case of inflammation of the sinuses of . 366
Uvula, removal of elongated, in case of obstinate cough . 559

Vagina, imperforation of the . 78
Vapour bath, Dr. Ridgway on the origin of the . . 215

PAGE
Varicose veins, new suggestion as to the treatment of . 409
Vein, obliteration of the left iliac 453
Veins, inflammation of, after labour . . . . 420
———, on inflammation of the 267
Venous inflammation, Mr. Chinnock's case of . . 176
Vertebræ, fracture of the, with curious symptoms . . 417
————, fractures of cervical 49
————, fracture of the dorsal 230
————, fracture of the cervical, 232
Violence, effects of, on the body after death . . . 464
Viscera, silver detected in the internal, after use of Argent. Nitr. . . . . 467

PAGE
Vitreous humor, ossification of . 554

Water, remarks on the effects produced by a shock of, by Dr. C. M. Clarke . . 458
Westminster Medical Society, petition of, respecting the supply of subjects for dissection 182
Wine, maturation of . . 180
Wollaston, Dr. death of . 185
Worms, analysis of Mr. Rhind's Treatise on Intestinal . 142
———, cure of . . . 148
———, formation of, in the intestines . . . 143
———, symptoms of intestinal 147

### CRITICAL ANALYSES.

Manuel of Midwifery, by Michael Ryan, m.d. &c. . . . 51
Practical Treatise on Parturition, by Samuel Ashwell, Surgeon; to which are appended two Papers, one containing some Remarks on Abdominal Surgery, the other on Transfusion, presented by Dr. Blundell . . . . . . . . . . ib.
Commentaries on Insanity. By Dr. Burrows . . . . . 61
Treatise on Intestinal Worms. By Mr. Rhind . . . 142
Letter on the Gibraltar Fever. By W. W. Fraser, Esq. . . 153
Observations on Cholera. By Dr. Christie . . . . . 159
Elements of General and Pathological Anatomy. By Dr. Craigie . 243
Letter on the Supply of Bodies for Dissection. By G. J. Guthrie, Esq. 260
Illustrations of the Diseases of the Breast. By Sir A. Cooper, Bart. &c. 323
Treatise on Diseases of the Chest. By Dr. Laennec . . . 338
Napoleon à Sainte Hélène. By J. Hereau . . . . . 354
Treatise on Obstructed and Inflamed Hernia, &c. By H. Stephens 424
Dissertation on a New System of Pulmonary Pathology. By P. P. P. Myddelton, m.d. . . . . . . . . 439
An Essay on the Use of the Nitrate of Silver. By J. Higginbottom 521
Further Observations on the Use of the Lancetted Stilettes in the Cure of permanent Strictures of the Urethra. By R. A. Stafford . 534
Synopsis of Modern Medical Jurisprudence. By J. S. Forsyth . 541

COLLECTANEA . . . *Pages* 75, 106, 264, 356, 449, 547
INTELLIGENCE . . . . 81, 182, 278, 371, 468, 564
METEOROLOGICAL REGISTER 90, 186, 282, 378, 474, 570

### NAMES OF AUTHORS,

*Of whose Works, Observations, &c. either a detailed Account, or more or less general View, is given in the present Volume.*

---

PAGE
ADAM, Dr. on the Mantis Tribe . . . . . . . 467
ANDREWS, Mr. on Lobelia Inflata . . . . . 558
ASHWELL, Mr. analysis of his Practical Treatise on Parturition . . 51
BAILLIE, Dr. M. cases of Paraplegia . . . . . . 278
BARCOME, Mr. case of Sudden Death from Rupture of the Aorta . 290
———— on Diseases in Demerara . . . . . 283
———— on Enlargement of the Scrotum . . . 289
BAUCHARD, Mr. on the employment of Slates for hastening the Maturation of Fruits . . . . . . . . . 180
BEDOR, Dr. case of extraordinary Growth of the Heart . . 547
BERNDT, Professor, on Camphor in Puerperal Mania . . . 556
BIGNARDI, Dr. case of Rupture of the Heart . . . . 548
BLANDIN, M. case of large Calculus in the Bladder . . . 237
BRIGHT, Dr. case of Extra-uterine Pregnancy . . . 50
BRODIE, Mr. case of Injury of the Head . . . 41
————, case of Fracture and Dislocation of a Lumbar Vertebra . 417
BOUGON, M. on the Treatment of Erysipelas Phlegmonodes by Compression 81
BURNETT, Mr. case of Diabetes . . . . . . 301
———— on Diseases of the Nails . . . . . . 91
BURROWS, Dr. analysis of his Commentaries on Insanity . . 61
CABARET, Dr. case of Imperforate Vagina . . . . . 78
CAFFORT, M. case of Hernia . . . . . . . 123
CAYOL, M. his case of Abscess of the Larynx and Sudden Death . 514
CHENEVIX, Mr. on Mesmerism . . . . . 219, 491
CHINNOCK, Mr. H. S. on Venous Inflammation . . . . 187
CHRISTIE, Dr. analysis of his work on Cholera . . . . 159
CHRISTISON, Dr. on the Effects of Violence on the Body after Death 464
CHURCHILL, Mr. J. M. Medical Botany . . . . . 282
CIVIALE, Dr. on Catarrhus Vesicæ . . . . . . 401
CLARKE, Dr. C. M. on the Effect of a Shock of Water in Neuralgia . 458
COCTEAU, M. on the Reproduction of the Crystalline Lens . . 449
COOPER, Sir ASTLEY, analysis of Illustrations of Diseases of the Breast 323
COOPER, Mr. B. S. case of Lithotomy, (the subject of the Libel in the Lancet) . . . . . . . . . . 127
COPELAND, Mr. case of Destruction of a Portion of the Spinal Cord . 267
COUPER, Dr. cases of Laryngitis . . . . . . 235
CRAIGIE, Dr. analysis of his Elements of General and Pathological Anatomy . . . . . . . . . . 243
DANCE, Dr. on Inflammation of Veins . . . . . 267
DAVY, Dr. on Nitrate of Silver as a Test for Vegetable and Animal Matter . . . . . . . . . . 464
———— on the Appearances of the Blood in Disease . . 550
DESCHARMES, M. P. on a Substitute for Coffee . . . 180
DOYLE, Dr. reflections on Colica Pictonum . . . . 285
DUFFIN, Mr. on Deformity of the Spine . . . . . 280
DUPUYTREN, M. on Treatment of Strictures of the Urethra . . 35
————, on Spots in the Cornea . . . . . 562
————, case of Strangulated Umbilical Hernia . . 321
————, case of Lithotomy . . . . . . 21
————, on Femoral Aneurism . . . . . . 173
EARLE, Mr. case of Wound of the Abdomen . . . . 48
FISCHER, M. on the Presence of Iron in Tin . . . . 464

PAGE
FLETCHER, Mr. case of Gleet cured by Sea-water Injections . . 275
FLOURENS, M. on the Central Points of the Nervous System . . 75
FORSYTH, Mr. analysis of his Synopsis of Modern Medical Jurisprudence 541
FRANCOIS, Dr. case of an Idiot of an Herbivorous Habit . . 276
FRASER, Mr. W. W. review of his Letter on the Gibraltar Fever . 153
GALY, M. on Corpuscles in the Eye . . . . . . 121
GANAL, M. on the Mode of making the Diamond . . . 181
GEISS, Dr. case of Double Uterus and Double Conception . . 178
GIBSON, Professor, case of Axillary Aneurism . . . . 314
GLIONNA, Dr. case of Suppuration of the Spleen . . . 77
GODRICH, Mr. his case of Hydrophobia . . . . 169
GOOD, Dr. J. M. his remarkable case of Preternatural Fœtation . 166
GORDON, Dr. G. A. on the State of Medicine in Italy . . . 475
GRANVILLE, Dr. A. B. case of Removal of a Tumor from the Nympha by a Jugum . . . . . . . . . 460
GREEN, Mr. on Mercurial Fumigations . . . . . 384
GUILLERY, M. case of Twins and Inflammation of the Womb . 420
GUNTHER, Dr. on Quinine and Digitalis in Consumption . . 557
GUTHRIE, Mr. on Inflammation of the Eyes . . . . 1
——— on the Supply of Bodies for Dissection . . . 260
———, his Introductory Lecture at the College of Surgeons . 373
HALL, Dr. MARSHALL, on Ergot of Rye in Leucorrhœa . . 379
HASSELL, Dr. on Chlorides in Sores . . . . . 561
HASTINGS, Dr. his case of Ischuria . . . . . 515
HAWKINS, Mr. C. case of Urinary Abscess . . . . 310
——— case of Wound of the Throat . . . 312
HERBST, Dr. E. F. G. on Cold Affusion in Poisoning by Prussic Acid 387
HEREAU, M. J. analysis of Napoleon à Sainte Hélène . . . 354
HERING, Professor, on the Velocity of the Motion of the Blood, &c. . 357
HIGGINBOTTOM, Mr. analysis of his Essay on the Use of Nitrate of Silver 521
HOLLAND, Dr. on Sudorific Medicines . . . . . 372
HOULTON, Mr. on the Emmenagogue Powers of Cheonopodium Olidum 278
HUTCHISON, J. Esq. case of Tumor removed from the Face . . 462
JACOBSON, Dr. case of Habitual Hemorrhage from the Mammæ . 554
JEWEL, Mr. S. W. case of Diseased Knee . . . . 291
JEWEL, Mr. G. case of Extra-uterine Fœtation . . . 36
——— Report on Midwifery . . . . . 381
JOHNSON, Dr. JAMES, case of Pneumo-thorax . . . . 360
KEY, Mr. his case of Calculus removed from the Bladder by the Urethral Forceps . . . . . . . . . 519
KREHN, M. case of Ossification of the Vitreous Humor . . 554
KRIMER, M. case of Lachrymal Calculus . . . . 78
——— on Diseases of the Heart from Onanism . . . 451
LAENNEC, Dr. analysis of his Treatise on Diseases of the Heart . 338
LAUBREIS, Dr. case of Pregnancy with Cancer of the Uterus . . 563
LAWRENCE, Mr. case of Fracture of the Vertebræ . . . 230
LEE, Dr. ROBERT, case of Inflammation of the Umbilical Vein . 455
——— on the Functions of the Intestines and Liver in the Fœtus 356
——— on Inflammation of the Spermatic Vein . . . 366
LEONARD, Mr. J. case of Perforation of the Stomach . . . 217
LISFRANC, M. case of Excision of the Uterus . . . . 174
LUGOL, Dr. case of Cancerous Tubercles . . . . 135
LYFORD, Mr. case of Spontaneous Cure of Femoral Aneurism . 412
MACPHERSON, Mr. G. case of Fungus Hæmatodes . . . 368
MAGLIARI, Dr. case of Strangulated Hernia reduced by external use of Belladonna . . . . . . . . . 558
MARSHALL, Mr. on Treatment of Burns . . . . . 297
MARTINET, M. on the use of Turpentine in Neuralgia . . . 203
MAYO, Mr. on Physiology . . . . . . . 280
MAYO, Mr. C. case of Aneurism of the Aorta . . . . 553

PAGE
MAURY, M. case of a Man who fancied he had a Serpent in his Belly . 450
MITIVIE, Dr. case of Extra-uterine Abdominal Pregnancy . . 275
MONTANCEIX, M. on Colica Pictonum treated by Alum . . 95
MOREAU DE JONNES, M. on the Epidemic of Gibraltar . . 82
MYDDELTON, Dr. on a new System of Pulmonary Pathology, analysis . 439
NAEGELE, Dr. on Absorption by the Uterus . . . . 176
NEWELL, Dr. case of Exfoliation of the Cuticle . . . 552
PARRISH, Dr. on Infantile Convulsions . . . . . 390
PATIN, M. on the use of the Acetate of Ammonia in Uterine Diseases 277
PHILIP, Dr. A. P. W. on Digestion . . . . . . 549
PHYSICK, Dr. case of Elongation of the Uvula . . . . 556
PINGRENON, Dr. case of Pulmonary Apoplexy . . . 359
POGGI, Dr. case of Tetanus . . . . . . . 133
PORTA, Dr. on the use of Tannin in Uterine Hemorrhage . . 456
PORTER, Dr. case of Bulimia . . . . . . . 553
QUARRIER, Dr. on Feigned Diseases of the Heart . . . 564
RAMSBOTHAM, Dr. F. cases of Malformation of the Heart . . 548
REINHARDT, Dr. on Borax in Diseases of the Skin . . . 557
REMOUD, M. on the Preparation of Grain and Seeds by Chlorine . 180
RENTON, Dr. case of Delirium Tremens . . . . 551
REYNAUD, M. case of Ovarian Tumor . . . . . . 306
——— case of Absence of the Menses in a Woman . . 452
——— case of Obliteration of the left Iliac Vein . . 453
RHIND, Mr. analysis of his Treatise on Worms . . . . 142
RICHERAND, Mr. case of Ligature of the external Iliac Artery . 273
RIDGWAY, Dr. on the Vapour Bath . . . . . 215
ROBERT, M. (Hôtel Dieu,) on Ulceration and Perforation of Duodenum 76
ROUX, M. case of Abscess in the Brain . . . . . 452
——— cases of Poisoning by the Berries of the Coriaria Myrtifolia 292
RYAN, Dr. analysis of his Manual on Midwifery . . . 51
SCHREYER, Dr. case of remarkable Disposition to Hemorrhage . 554
SCHUTTE, Dr. on the Cure of Fungus Hæmatodes by Alum, &c. . 273
SHEPPARD, Mr. case of Fracture of the Leg . . . . 47
SIMPSON, Dr. case of Hernia, six inches of Intestine removed . . 560
SMITH, Mr. J. on Delirium Tremens . . . . . . 9
SOEMMERING, M. S. T. on the Conformation of the Human Stomach . 166
SOUQUET, M. case of chronic Ptyalism . . . . . 269
SPEER, Dr. on Diseases on board the Essex . . . . 197
SPERANZA, Dr. on the external use of Quinine . . . . 557
STAFFORD, Mr. analysis of his Essay on Strictures . . . 534
STEGGALL, Dr. Introduction to Botany . . . . . 281
STEPHENS, Mr. on Obstructed and Inflamed Hernia, analysis . . 424
STEPHENSON, Dr. Medical Botany . . . . . 282
STRETTEN, Dr. case of Perforation of the Duodenum . . . 43
SYME, Mr. on Exfoliation from the Bones of the Pelvis . 410, 559
TRAVERS, Mr. cases of Injuries of the Head . . . . . 38
TWINING, Mr. W. case of Fungus of the Eye . . . . 141
——— on Diseases of the Spleen . . . . 169
TYRRELL, Mr. case of Fracture of the fourth Cervical Vertebra . 232
WALLER, Mr. Report on Midwifery . . . . . 115
WALSH, Mr. J. on Diseases in Hindostan . . 25, 102, 207
WEBSTER, Dr. J. on East-Indian Opium . . . . 511
WEDEMEYER, Dr. case of Silver detected in the internal Viscera . 467
WICKHAM, Mr. case of Lithotomy . . . . . 131
WILSON, Dr. J. on the Association of Turpentine and Mercury . 501
WOOD, Mr. case of Dropsy of the Pericardium . . . . 406

J. and C. Adlard, Printers, Bartholomew Close.

